# Insights into the intestinal microbiota of *Exopalaemon annandalei* and *Exopalaemon carinicauda* in the Yangtze River estuary

**DOI:** 10.3389/fcimb.2024.1420928

**Published:** 2024-10-09

**Authors:** Jiahao Wang, Guangpeng Feng, Zhiqiang Han, Tao Zhang, Jinhui Chen, Jianhui Wu

**Affiliations:** ^1^ East China Sea Fisheries Research Institute, Shanghai, China; ^2^ Zhejiang Ocean University, Zhoushan, China; ^3^ Shanghai Aquatic Wildlife Conservation Research Center, Shanghai, China; ^4^ Shanghai Monitoring Station of Aquatic Biological Resources in the Yangtze River Basin, Shanghai, China

**Keywords:** gut microbiome, *Exopalaemon annandalei*, *Exopalaemon carinicauda*, high-throughput sequencing, Yangtze River estuary

## Abstract

Summary: The gut microbiota plays a crucial role in food webs, carbon cycling, and related elements. *Exopalaemon annandalei* and *Exopalaemon carinicauda* are two important forage species in the Yangtze River estuary with extremely similar living habits and morphological characteristics. Exploring the microorganisms in the guts of these two shrimp species can help us understand the survival status of forage species and gut microbiota in the Yangtze River estuary. Therefore, this study analyzed the similarities and differences in the intestinal flora of *E. annandalei* and *E. carinicauda* through high-throughput sequencing of 16S rRNA gene amplicons. The results showed that the dominant bacteria in the intestinal flora of *E. annandalei* and *E. carinicauda* at the phylum level were Proteobacteria and Firmicutes, respectively. At the genus level, the intestinal flora had higher concentrations of *Psychrobacter*, *Bacillus*, *Pseudomonas*, *Acinetobacter*, and *Macrococcus*. In both shrimp species, the contents of *Acinetobacter* and *Macrococcus* were higher in spring than in winter. The most important potential functions of the intestinal microbiota were amino acid metabolism and purine metabolism. Additionally, the functions of metabolism and diseases in the intestinal microbiota of *E. annandalei* were greatly influenced by the season. Furthermore, the experimental results indicated that a lower ratio of *Firmicutes* to *Bacteroidetes* was associated with a larger body weight in shrimp. Overall, this study provides a theoretical reference for understanding the intestinal bacterial community of shrimp in estuaries and the healthy cultivation of *E. annandalei* and *E. carinicauda*.

## 1 Introduction

The intestines of shrimp are inhabited by a vast number and diverse array of bacteria. The gut of shrimp is home to a large and diverse number of bacteria that can affect the healthy growth, disease, and feeding of shrimp. Studying the intestinal microbiota can assist in revealing the operational patterns of the shrimp intestine. And laying a foundation for a deeper understanding of the relationship between shrimp and microorganisms. At the same time, winter and spring are the seasons when the intestinal microbiota of shrimp changes greatly (Tang et al., 2014). Therefore, it is necessary to study the intestinal microbiota to reveal the operation mode of shrimp gut. The common dominant genera in the intestinal flora of shrimp include *Photobacterium*, *Vibrio*, *Aeromonas* and *Pseudomonas* (Yasuda and Kitao, 1980; Dempsey et al., 1989). Additionally, *Pseudomonas*, *Brevundimonas*, *Ralstonia*, *Caulobacter*, *Lactococcus*, and *Sphingomonas* have also been identified as dominant species in the intestines of some shrimps (Kim et al., 1997; Lin et al., 2022). The structure of the gut microbiota has a significant impact on the growth and development of shrimps. In terms of nutritional functions, the gut microbiota of shrimps not only synthesizes nutrients but also affects the metabolism of shrimps by synthesizing amino acids and unsaturated fatty acids (Semova et al., 2012; Zhang et al., 2014). Furthermore, bacteria can sometimes serve as a food source for shrimps (Yasuda and Kitao, 1980). In terms of aiding digestion, secretions from the gut microbiota, such as vitamins, amino acids, and highly unsaturated fatty acids, can all be utilized by the host (Almansa et al., 2012). In terms of immune function, nutrients produced by the gut microbiota can provide energy for the intestinal mucosa, indirectly promoting mucosal repair and growth (Zhao et al., 2013; Lee and Hase, 2014; Antonissen et al., 2016; Kubasova et al., 2019; Ocejo et al., 2019). Additionally, probiotics can significantly enhance the immunity of shrimps (Aguilera-Rivera et al., 2014; Zokaeifar et al., 2014).

Currently, high-throughput sequencing technology is widely utilized in the analysis of gut microbiota structure in aquatic organisms. By comparing the sequencing results with databases, the classification of the gut microbiota can be accurately determined down to the species level (Zhou et al., 2011). Compared to conventional detection methods, high-throughput sequencing technology offers advantages such as high sensitivity (able to capture low-frequency DNA sequences), high sequencing throughput, high accuracy, and cost-effectiveness. It is one of the effective techniques for studying the structure of the gut microbiota today and in the near future (Backhed et al., 2004; Backhed et al., 2005; Ley et al., 2005). Currently, high-throughput sequencing technology has been applied in the study of the gut microbiota of various shrimp species, including *Procambarus clarkia* (Shui et al., 2020), *Macrobrachium rosebergii* (Xu et al., 2023) and *Penaeus monodon* (Rungrassamee et al., 2014). The high-throughput sequencing has the effectiveness and potential in understanding the composition and function of the gut microbiota in shrimp.

*E. annandalei* and *E. carinicauda* are widely distributed in China. As important bait species in the Yangtze River estuary (Pei et al., 2017), they are an integral part of the resources in the estuary (Geng et al., 2022). The study of the intestinal microbiota of *E. annandalei* and *E. carinicauda* aims to provide insights into the changes in aquatic biological resources in the region. This research also serves to further enhance our understanding of the structural and functional information of the aquatic ecosystem in the area, providing a theoretical basis for promoting ecological restoration efforts. Additionally, it offers literature references for shrimp farming and introduces a novel technical approach for analyzing the feeding habits of aquatic organisms in the Yangtze River estuary.

Given the ecological conditions of the Yangtze River estuary, this study selected the dominant species, *E. annandalei* and *E. carinicauda*, as the research subjects. By utilizing 16S rRNA primers, we analyzed the gut microbiota diversity of these two shrimp species in winter and spring in the Yangtze River estuary, and predicted their functional roles.

## 2 Materials and methods

### 2.1 Sample collection

**Figure 1:**
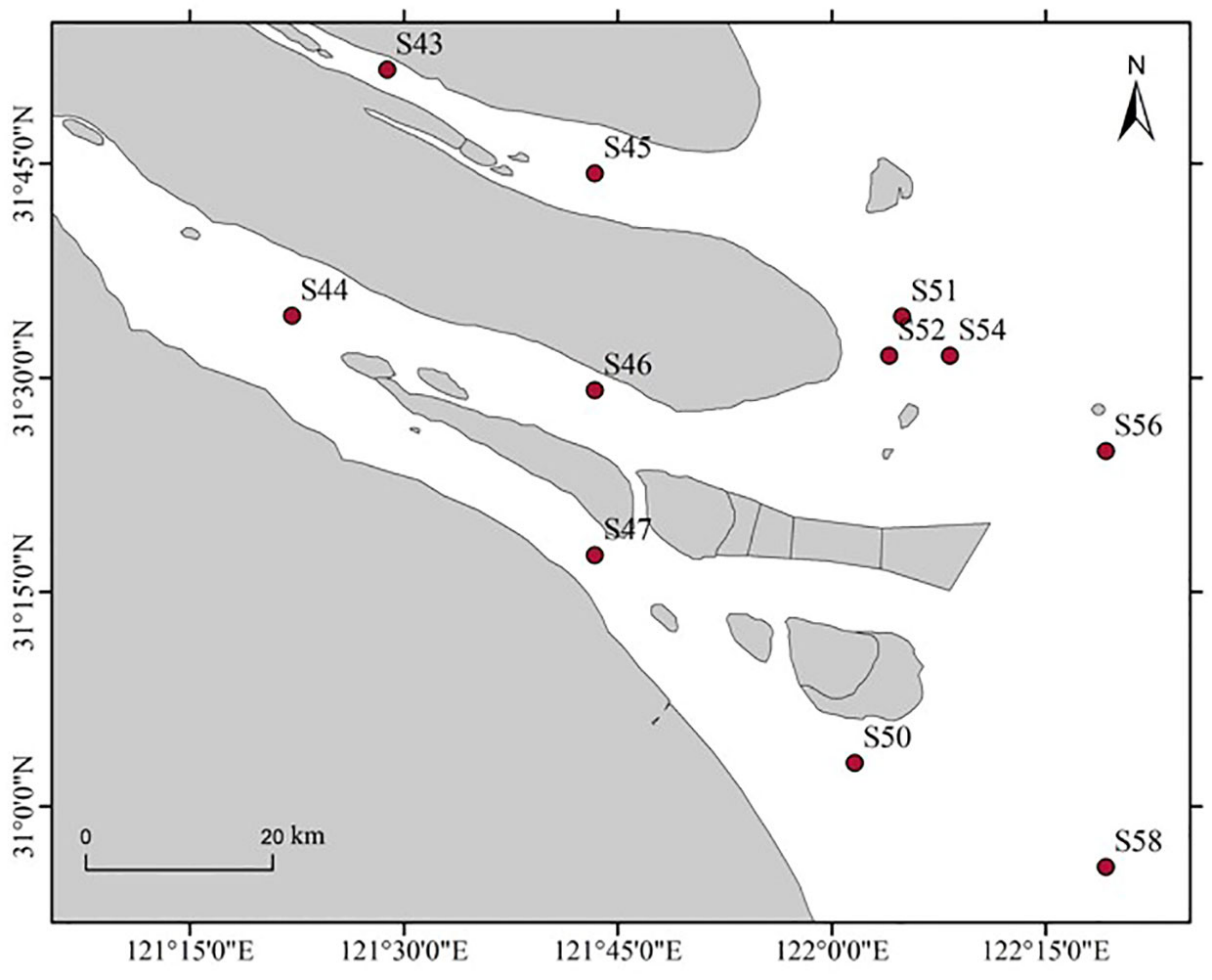
Survey sampling map of shrimp in the Yangtze River estuary.

**Figure 2:**
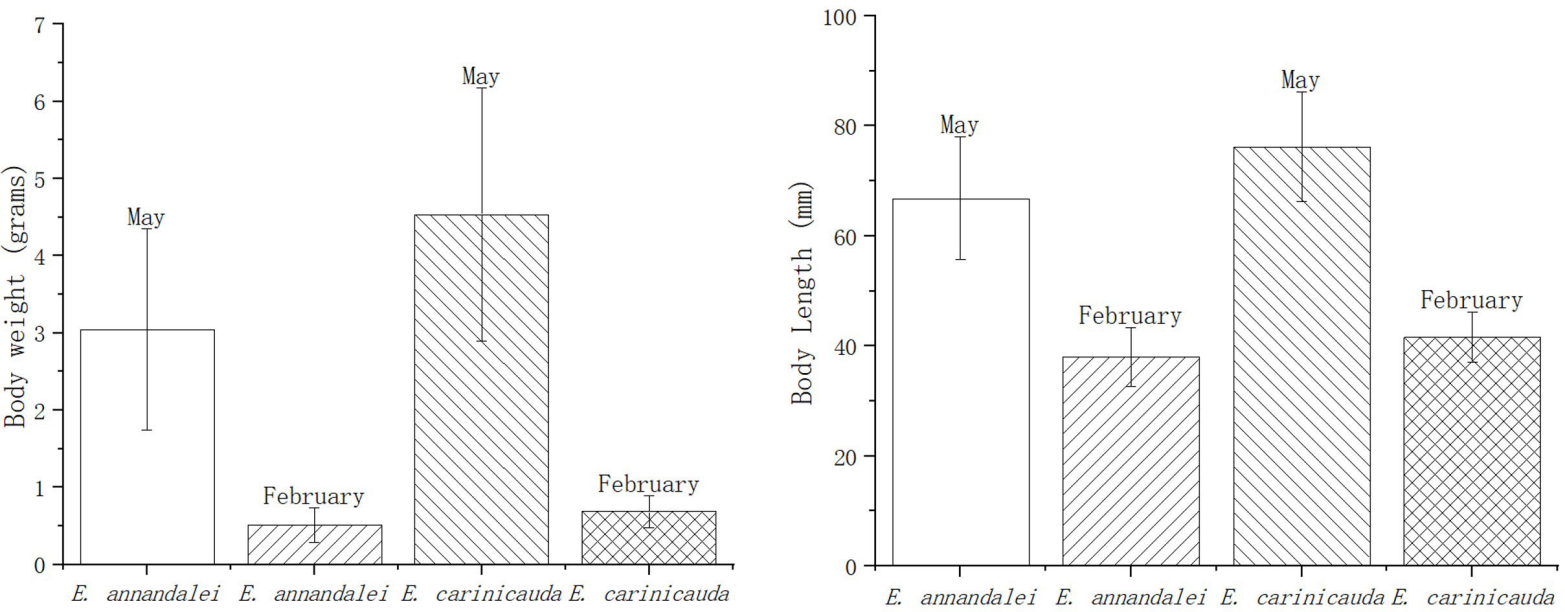
Histogram of average shrimp body length and weight caught at each point.

**Figure 3:**
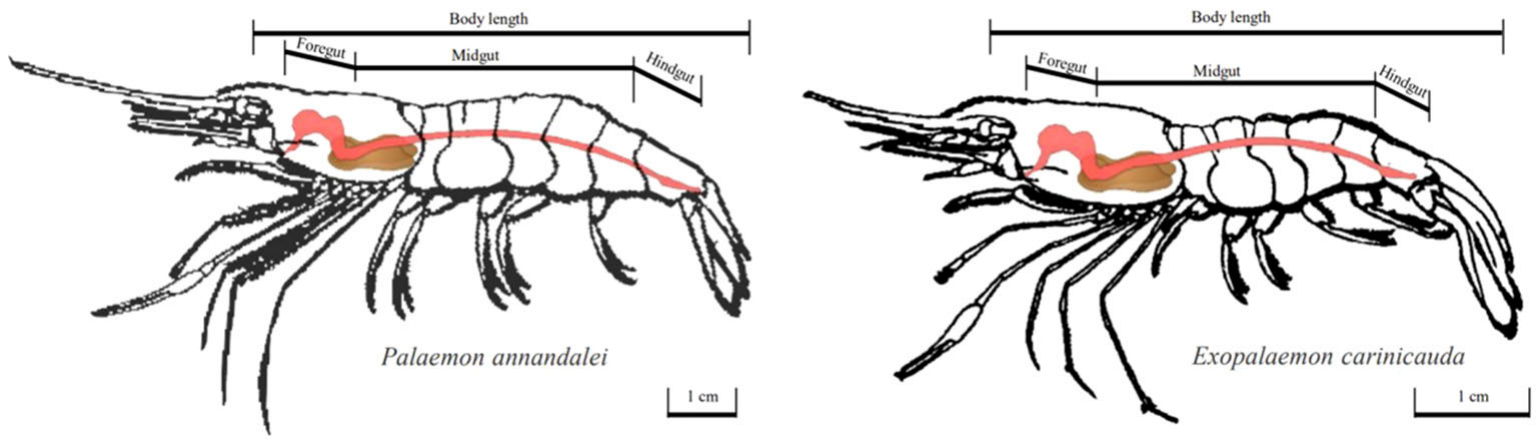
Schematic diagram of the shape of the shrimp.

In February and May 2023, *E. annandalei* and *E. carinicauda* in the Yangtze River estuary (121°33′E~122°14.5′E、31°46′N~31°24′N) were collected by single-bag otter trawls (**Figure 1**). The body length of *E. annandalei* ranged from 22 to 42 mm, and the body weight ranged from 0.173 to 1.242g The body length of *E. carinicauda* ranged from 28 to 71 mm (**Figure 2**), and the body weight ranged from 0.4216 to 7.1278 g (**Figure 3**). 20 shrimps were randomly selected at each sample. Four experimental groups are set up as follows: intestinal flora of *E. annandalei* in winter with 7 parallel samples; intestinal flora of *E. annandalei* in spring with 7 parallel samples; intestinal flora of *E. carinicauda* in winter with 3 parallel samples; intestinal flora of *E. carinicauda* in spring with 3 parallel samples. For each parallel sample, intestines of 20 shrimps are selected for mixing.

### 2.2 DNA extraction and PCR amplification

DNA was extracted from the intestine of shrimp using the E.Z.N.A.^®^ Soil DNA Kit (Omega Bio-tek, Norcross, GA, U.S.). The V4-V5 region of the bacterial 16S rRNA gene was amplified through PCR. Using the primers 515F 5’-barcode-GTGCCGCCAGCMGCCGG-3’ and 907R 5’-CCGTCAATTCMTTTRAGTTT-3’, where the barcode represents a unique eight-base sequence for each sample. Three replicate PCR reactions were performed in a 20 μL mixture containing 4 μL of 5× FastPfu buffer, 2 μL of 2.5 mM dNTPs, 0.8 μL of each primer (5 μM), 0.4 μL of FastPfu polymerase, and 10 ng of template DNA. All samples were processed in accordance with the official experimental conditions, with each sample replicated three times. For the study of bacterial community structure, PCR amplification was performed using primers targeting the V3-V4 region of the 16S rRNA gene, specifically the 341F primer (upstream) and the 806R primer (downstream).

### 2.3 Processing of sequencing data and statistical analysis

The purified PCR product was quantified by Qubit^®^3.0 (Life Invitrogen) and each of the 24 amplicons with different barcodes was mixed equally. The amplicon libraries were sequenced using the Illumina MiSeq platform (Shanghai BIOZERON Co., Ltd.) with a standard dual-end sequencing protocol (2×250).After initial filtering and removal of chimeras, the raw sequencing data were processed to obtain valid data. Concurrently, the self-sequences of the two species of shrimp were excluded, and manual verification was conducted in accordance with the distribution information of aquatic organisms across various ecological types in the Yangtze River estuary (Fang, 2011). Clustering OTUs by QIIME software, bar charts were generated to depict the diversity of bait organisms and the relative abundances of different species. Based on the functional prediction results from PICRUSt2, the relative abundances of KEGG pathways in each sample or group were analyzed at the level 1, level 2, and level 3 categories. Subsequently, KEGG pathway abundance statistical charts were generated to visualize these data. Using the Spearman correlation method, a coexistence network of microbial communities in the study samples was constructed. Calculate the Spearman correlation between the abundances of every two OTUs and calibrate the results with FDR. Only results with a correlation coefficient greater than 0.8 and a calibrated p-value less than 0.05 were retained, and a coexistence network graph was plotted accordingly.

## 3 Results and discussion

### 3.1 Bacterial communities in two shrimp intestinal

Based on the analysis of 20 samples, the top ten bacterial flora with the highest content in the intestinal tract of *E. annandalei* and *E. carinicauda* are *Psychrobacter*, *Acinetobacter*, *Macrococcus*, *Bacillus*, *Vibrionimonas*, *Lysinibacillus*, *Bradyrhizobium*, *Pantoea*, *Mycobacterium* and *Carnobacterium*. The top five bacterial flora with the highest content in the intestinal tract of *E. carinicauda* in February are *Psychrobacter*, *Bacillus*, *Vibrionimonas*, *Pseudomonas*, and *Staphylococcus*. In May, the top five bacterial flora with the highest content in the intestinal tract of *E. carinicauda* are *Psychrobacter*, *Acinetobacter*, *Macrococcus*, *Vibrionimonas*, and *Bradyrhizobium*. In February, the top ten bacterial flora with the highest content in the intestinal tract of *E. annandalei* are *Psychrobacter*, *Macrococcus*, *Vibrionimonas*, *Mycobacterium*, and *Pantoea*. In May, the top five bacterial flora with the highest content in the intestinal tract of *E. annandalei* are *Acinetobacter*, *Psychrobacter*, *Macrococcus*, *Bradyrhizobium*, and *Mycobacterium*.

### 3.2 Cluster analysis

**Figure 4:**
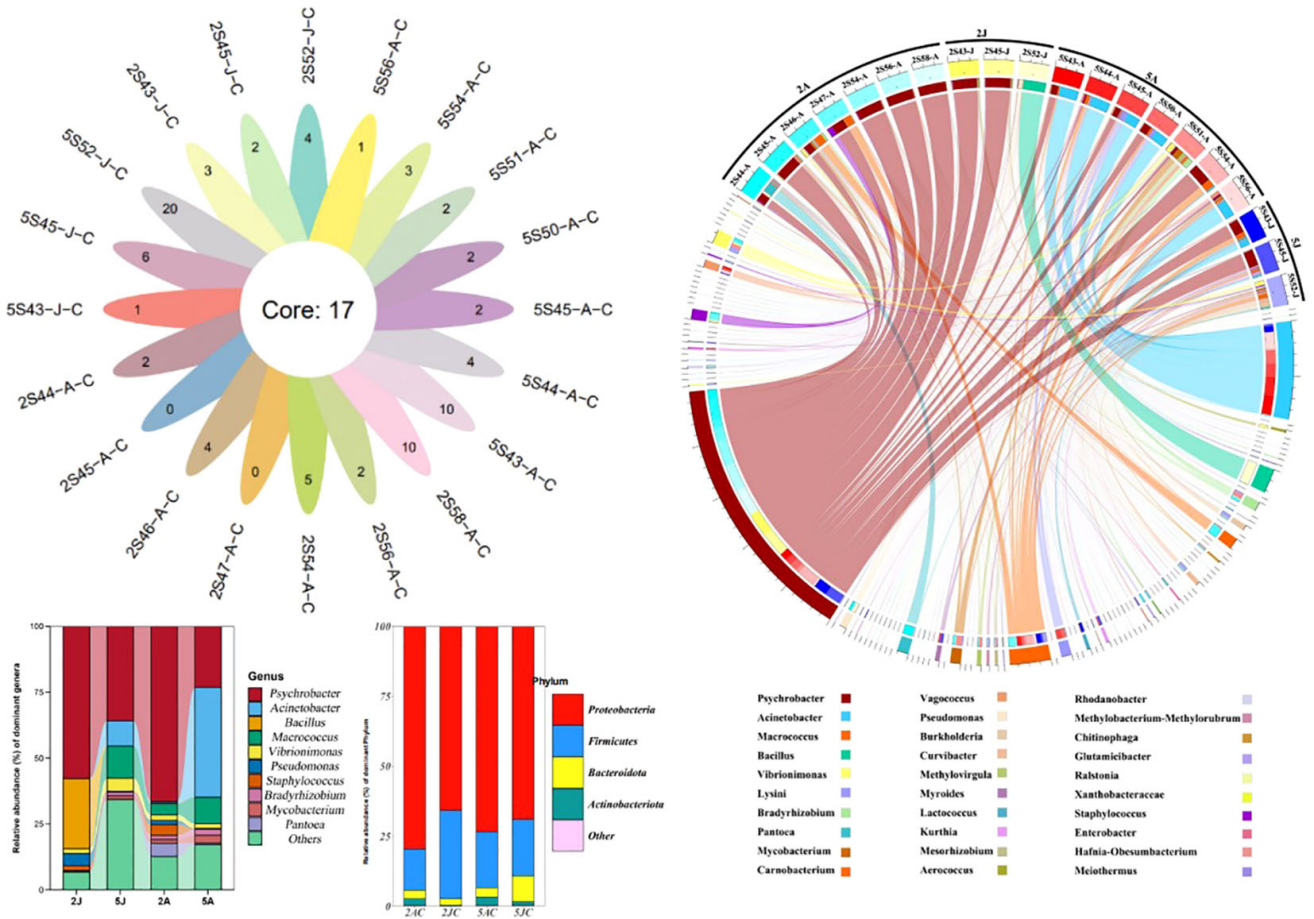
Analysis diagram of intestinal microbiota composition. To optimize the visual effect, components with an abundance below 0.1% can be combined as “other genera” for display in the graph. In the circos species analysis chart, the top 30 most abundant bacterial communities among all detected microbiota are shown. Group 2A represents *Exopalaemon annandalei* collected in February (Winter), Group 2J represents *Exopalaemon carinicauda* collected in February (Winter), Group 5A represents *Exopalaemon annandalei* collected in May (Spring), and Group 5J represents *Exopalaemon carinicauda* collected in May(Spring), same as above. Different shades of the same color are used to indicate different sampling locations within the same group.

Through cluster analysis of the four groups of 20 samples, a total of 541 OTUs were obtained. As shown in Venn (**Figure 4**), the OTU sequences detected in the intestinal microbiota of *E. carinicauda* in February, *E. carinicauda* in May, *E. annandalei* in February, and *E. annandalei* in May were 193, 380, 391, and 432, respectively. Among them, 137 OTU sequences were shared by all four groups, accounting for 25.3%. Additionally, 330 OTUs were unique to *E. annandalei*, accounting for 57.46%, and 153 OTUs were unique to *E. carinicauda*, accounting for 28.259%.

### 3.3 Functional prediction

Based on the KEGG functional prediction, the functions of the intestinal microbiota are primarily concentrated in metabolism, specifically in energy metabolism, carbohydrate metabolism, and amino acid metabolism, accounting for 70.19%, 14.95%, and 3.67% respectively. According to the bubble chart of KEGG level 1 inter-group differences, there are differences in the intestinal flora functions of the two species of shrimp in two aspects: metabolism and human diseases. There are also differences in the intestinal flora functions of *E. annandalei* in February and May, while there are no significant differences in the intestinal flora functions of *E. carinicauda* sinensis in the two months.

Further analysis suggests that the function of human pathogens pneumonia may be lower in the intestinal flora of *E. annandalei* compared to that of *E. carinicauda*. Additionally, the increase in the function of aromatic compound degradation and the decrease in the function of nitrate reduction in both species of shrimp in May may have contributed to these observed differences.

### 3.4 Network analyze

According to the Co-occurrence network comparison chart between winter and spring for the two types of shrimp, February *Exopalaemon annandalei* network had 60 nodes and 587 edges, February *E. carinicauda* network had 36 nodes and 342 edges, May *Exopalaemon annandalei* network have 76 nodes and 827 edges, May *E. carinicauda* network have 69 nodes and 1038 edges. According to previous research (Zeng QC, An SS, Liu Y, Wang HL, Wang Y (2019) Biogeography and the driving factors affecting forest soil bacteria in an arid area. Sci Total Environ 680:124–131), the higher the proportion of competition between bacterial species, the better the stability of the network under environmental interference. In the spring when bacterial richness and diversity are high, the interaction between bacteria is also stronger, further complicating the network. Therefore, the intestinal microbiota results of the two shrimp species were susceptible to seasonal variation.

## 4 Discussion

### 4.1 Analysis of major bacterial populations in the gut of two shrimps

**Figure 5:**
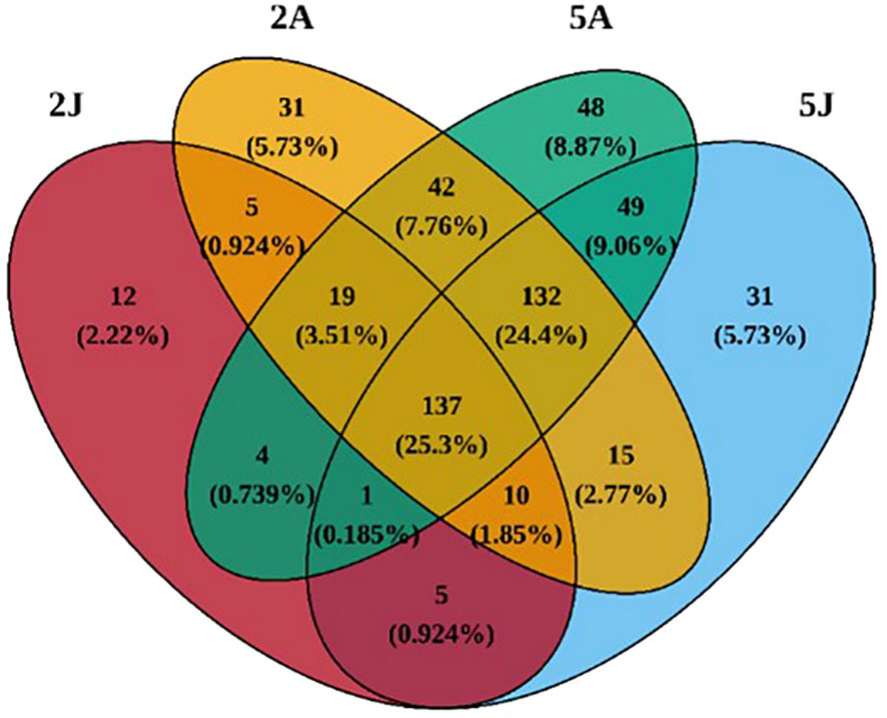
Venn of intergroup core OTUs.

In this study, we identified Proteobacteria and Actinobacteriota as the dominant bacterial phyla in the intestines of two species of shrimp based on OTU abundance. They are one of the most widely distributed bacterial phyla in freshwater bodies and sediments (Kendrick and Hyndes, 2005). In addition, Proteobacteria perform functions and may also have cellulose and agar degradation as well as nitrogen fixation in the rectum of shrimp (Li et al., 2018). The Actinobacteriota phylum can improve the digestion and immunity of shrimp. This bacterium has been reported to be a potential probiotic in aquaculture ponds (Costantini et al., 2017). Bacteria of Bacteroidota mainly exist in pond sediments and can effectively degrade refractory organic matter in wastewate (Tzeng et al., 2015). In February (winter) and May (spring), the dominant genus shared by the intestinal microbiota of *E. carinicauda* and *E. annandalei* was *Psychrobacter*. The proportion of *Psychrobacter* in *E. carinicauda* decreased from 57.72% in winter to 35.59% in spring, while the proportion of *Psychrobacter* in *E. annandalei* decreased from 65.88% in winter to 22.53% in spring (**Figure 5**).

Research has shown that *Psychrobacter* can help improve the local microbial diversity in the gastrointestinal tract of Escherichia coli-like bacteria (Yang et al., 2011). In addition, *Psychrobacter* produces chitinase and protease, which are extracellular enzymes secreted by bacteria. These enzymes exhibit catalytic activity under acidic conditions (pH 2–5) and within a temperature range of 15 to 30°C. This genus is a potential probiotic that can promote the digestion of shrimp (Moss et al., 2000). Previous studies have shown that *Psychrobacter* can effectively promote the growth, immunity, antioxidant capacity, and disease resistance of fish, indicating that the intestinal health of the two species of shrimp in winter is better than that in spring (Bakermans et al., 2006; Zhao et al., 2018). In future studies, it is recommended to simultaneously evaluate environmental physicochemical factors and microbial community composition to assist in analyzing the characteristics of the intestinal microbiota of benthic animal communities. *Psychrobacter* can also be used as an indicator to judge the health of shrimp.

### 4.2 Relationship between intestinal microbiota and seasons in two shrimp species

**Figure 6:**
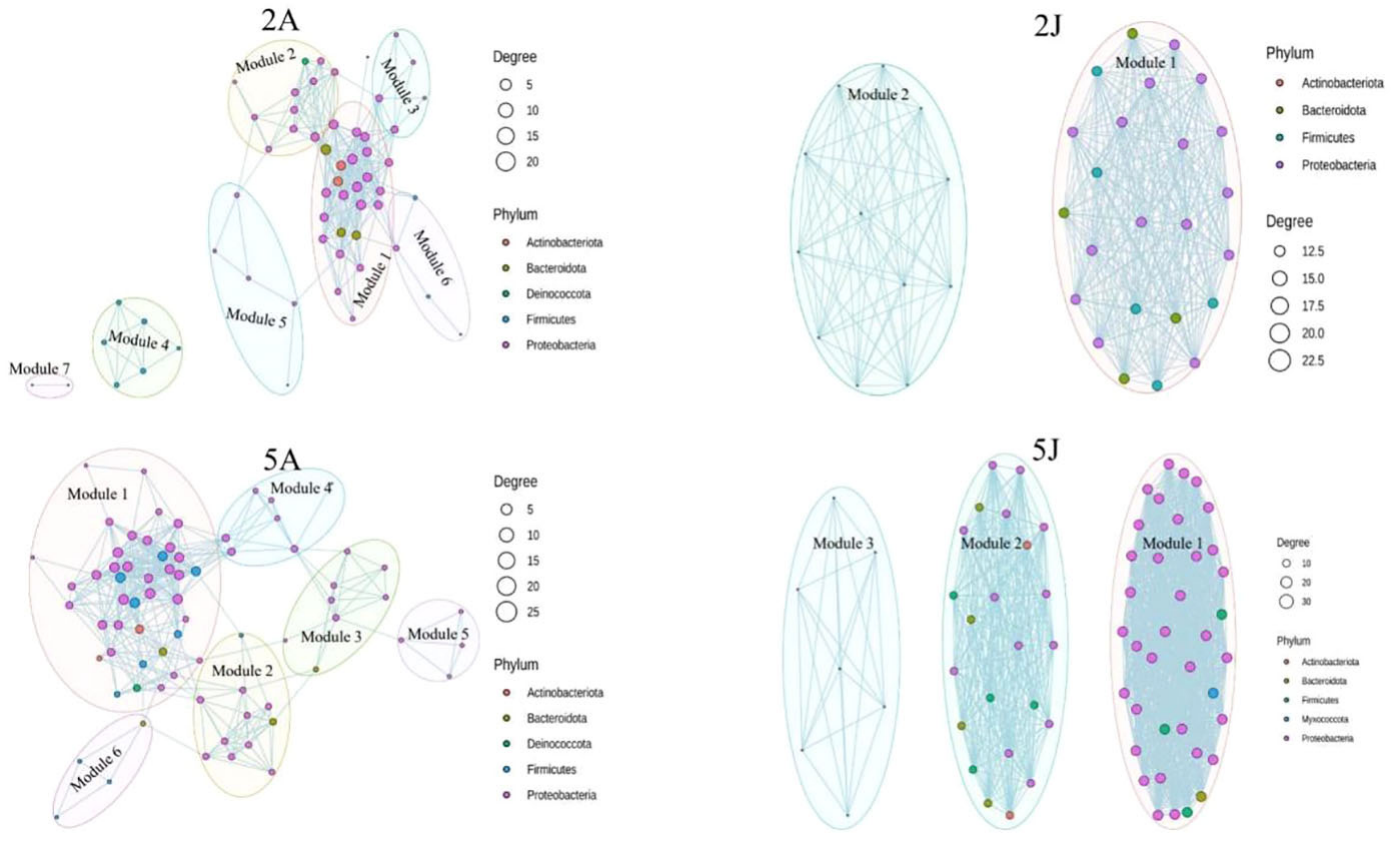
Co-occurrence network diagram.

At the phylum level, there is little difference in the intestinal microbiota of *E. annandalei* between winter and spring, which is mainly dominated by Proteobacteria (Sekirov et al., 2010). At the same time, the relative abundance of *Firmicutes* in the intestinal microbiota is also relatively high. In contrast, the intestinal microbiota of *E. carinicauda* exhibits significant differences between winter and spring. At the phylum level, the number of *Firmicutes* in the intestinal microbiota of *E. carinicauda* is higher in winter than in spring, while the number of *Bacteroidota* is much lower in winter than in spring. Research has shown that the ratio of Firmicutes to *Bacteroidota* is directly related to body weight (Ley et al., 2006). The increase in the number of *Firmicutes* directly leads to an increase in the number of lipid droplets, thereby proportionally enhancing the absorption of fatty acids (Semova et al., 2012). In addition, the ratio of *Firmicutes* to *Bacteroidota* continues to change with different life stages and salinity levels of shrimp (Mariat et al., 2009; Fan et al., 2019). Since the body weights of both species of shrimp in spring are greater than those in winter, it can be concluded that a smaller ratio of *Firmicutes* to *Bacteroidota* corresponds to a larger body weight of shrimp (**Figure 6**) (Arora et al., 2011).

At the genus level, the proportion of *Acinetobacter* and *Macrococcus* in the intestinal microbiota of *E. annandalei* increased from less than 0.1% in winter to 38.23% and 9.85%, respectively. In contrast, the proportion of *Acinetobacter* and *Macrococcus* in the intestinal microbiota of *E. carinicauda* increased from less than 0.1% in winter to 9.04% and 12.13%, respectively. Additionally, there was a significant decrease in the proportion of Pseudomonas in the intestines of both *E. annandalei* and *E. carinicauda*. The *Macrococcus* genus, a *Bacterial* genus, was found in the intestinal microbiota of both *E. carinicauda* in February and *E. annandalei* in February and May. Research has shown that *Macrococcus* can digest and decompose high-carbohydrate, low-protein diets (Villasante et al., 2022). This genus can produce enzymes such as amylase and protease, which contribute to the digestion of carbohydrates in the intestine (Kloos et al., 1998). *Acinetobacter* is a common genus in the intestinal microbiota of both *E. annandalei* and *E. carinicauda* in May. Studies have shown that *Acinetobacter* can metabolize lactic acid and acetic acid produced by cytoplasmic secretions (Savard et al., 2023). It is speculated that the abundance of prey organisms in the estuary of the Yangtze River in spring leads to an increase in the feeding rate of shrimp, resulting in an increase in cytoplasmic secretions in the intestines of shrimp. Therefore, the increase in cytoplasmic secretions may have contributed to the increase in the relative abundance of *Acinetobacter* in the samples. Research has indicated that most *Pseudomonas* species play an important role in wastewater treatment and oil spill cleanup (Kersters et al., 2006). Based on this, it is speculated that the pollution levels from wastewater and oil spills in the estuary of the Yangtze River are lower in spring than in winter, which may be related to the water volume of the Yangtze River (Warwick and Clarke, 1991). It can be inferred that both species of shrimp consume more carbohydrates and proteins in spring than in winter. Based on this, it can be speculated that the Yangtze River Estuary consumes more carbohydrates and proteins in spring, which leads to the increase of *Acinetobacter and Macrococcus* in both intestines.

### 4.3 Functions of the intestinal flora of two species of shrimp

**Figure 7:**
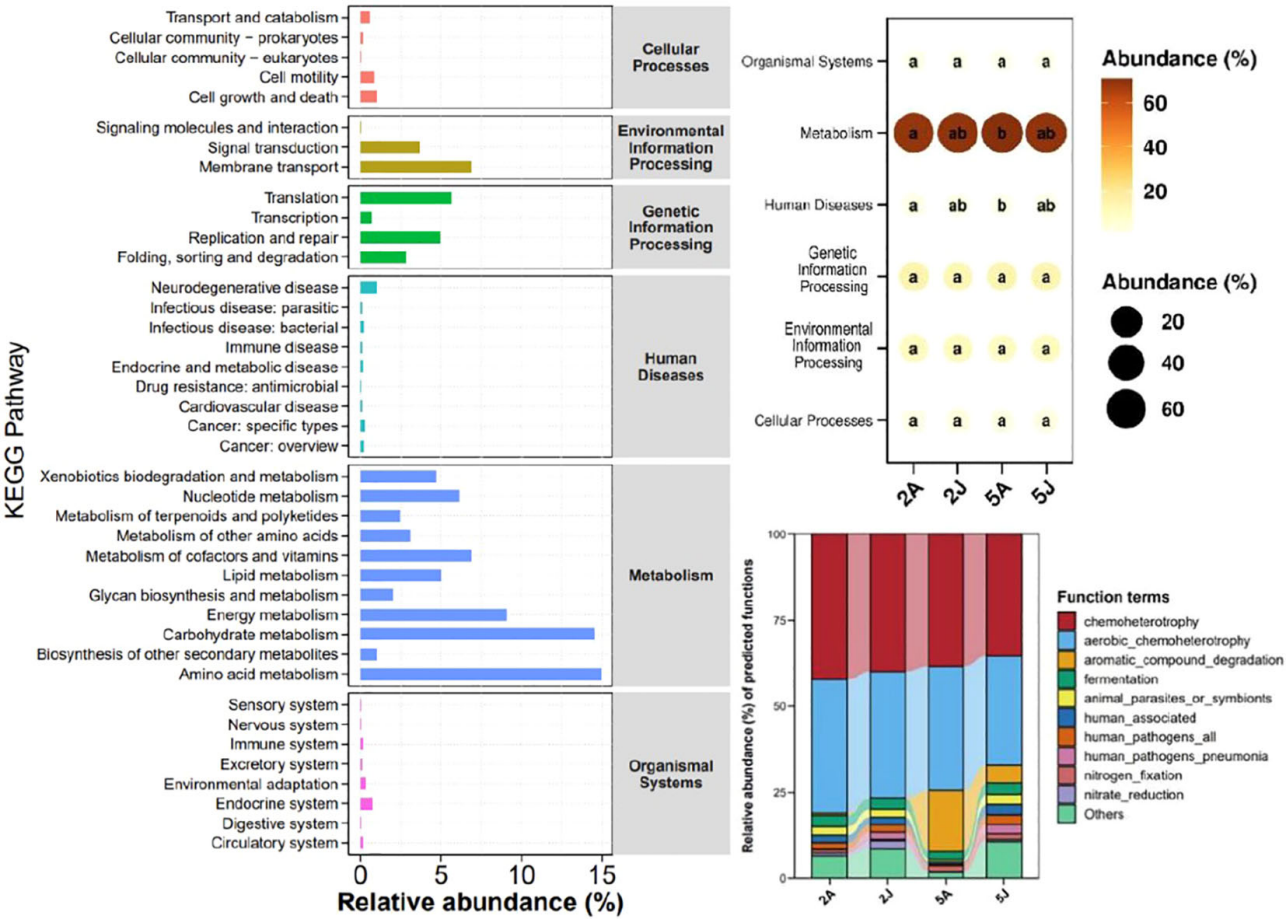
KEGG pathway abundance statistics chart.

Intestinal microbiota play a crucial role in metabolic processes by providing direct substrates for memory cells (Block and Carey, 1985; Bäckhed, 2011. The prediction of KEGG pathways in this study indicates that the intestinal microbiota of *E. carinicauda* and *E. annandalei* play crucial roles in several aspects of metabolism, including xenobiotics biodegradation and metabolism, nucleotide metabolism, metabolism of terpenoids and polyketides, metabolism of other amino acids, metabolism of cofactors and vitamins, lipid metabolism, glycan biosynthesis and metabolism, energy metabolism, carbohydrate metabolism, and amino acid metabolism. Meanwhile, they also play significant roles in Signal Transduction and Membrane Transport, which are two aspects of Environmental Information Processing, as well as Translation, Replication and repair, and Folding, sorting and degradation, which are three aspects of Genetic Information Processing (**Figure 7**).

Through the functional analysis of intestinal microbiota, we can infer that the intestinal microorganisms of *E. carinicauda* and *E. annandalei* have specific feeding habits, and they may consume foods containing amino acids and purines. Such feeding habits may have significant impacts on the growth and health of shrimp. Amino acids are essential nutrients for organisms, playing a crucial role in protein synthesis. Purines are organic compounds in organisms that participate in the synthesis of biological molecules such as DNA and RNA, which are crucial for the normal physiological functions of organisms. Therefore, this feeding habit of intestinal microbiota provides crucial substrates for the growth and health of shrimp (Shen et al., 2015; Zhao et al., 2023).

The research results indicate that there are differences in the function of intestinal microbial communities between *E. annandalei* and *E. carinicauda* in terms of metabolism and disease. The intestinal microbial community function of *E. annandalei* varies with seasonal changes, while the function of the intestinal microbial community of *E. carinicauda* is less affected by seasonal changes. Therefore, in aquaculture, more attention should be paid to the impact of seasonal changes on *E. annandalei* compared to *E. carinicauda*. Additionally, appropriate bacteria or bait can be introduced to promote the metabolism and disease resistance of these shrimp species. By understanding the specific microbial communities and their functions in different shrimp species, aquaculture practices can be optimized to improve the health and productivity of these economically important animals.

## 5 Conclusion

In summary, protein metabolism and purine metabolism are the main functions of the intestinal microbiota in both shrimp species, and both shrimp species consume more carbohydrates and protein foods in spring than in winter. There are similarities and differences in the intestinal microbiota of the two shrimp species. The intestinal microbiota function of *E. annandalei* is greatly influenced by seasons, while the intestinal microbiota function of *E. carinicauda* is not affected by seasons. In addition, it was also found that the smaller the ratio of *Firmicutes* to *Bacteroidetes*, the greater the body weight of the shrimp. Future research should focus on the functions of the shrimp intestinal microbiota and the impact of environmental factors on the intestinal microbiota, providing information for establishing sustainable biological management strategies.

## Data availability statement

The raw data supporting the conclusions of this article will be made available by the authors, without undue reservation.

## Ethics statement

Ethical approval was not required for the studies on animals in accordance with the local legislation and institutional requirements because only commercially available established cell lines were used.

## Author contributions

JWa: Writing – original draft. GF: Writing – review & editing. ZH: Writing – review & editing. TZ: Writing – review & editing. JC: Conceptualization, Writing – review & editing. JWu: Writing – review & editing.
